# Pathological Allostery in ADAMTS13: Autoantibody-Induced Modulation and Its Role in Immune Thrombotic Thrombocytopenic Purpura (iTTP)

**DOI:** 10.3390/ph19081186

**Published:** 2026-07-29

**Authors:** Madison Gil, Konstantine Halkidis

**Affiliations:** 1 Department of Pathology and Laboratory Medicine, The University of Kansas Medical Center, Kansas City, KS 66160, USA; mshay@kumc.edu; 2 Department of Internal Medicine, Division of Hematologic Malignancies and Cellular Therapeutics, The University of Kansas Medical Center, Kansas City, KS 66160, USA; 3 Institute for Reproductive and Developmental Sciences, The University of Kansas Medical Center, Kansas City, KS 66160, USA

**Keywords:** allostery, thrombotic thrombocytopenic purpura, ADAMTS13, antibodies, inhibition, thermodynamics, mechanism

## Abstract

**Background/Objectives**: ADAMTS13 is a plasma metalloprotease that cleaves von Willebrand Factor (vWF), a multimeric glycoprotein involved in platelet recruitment during primary hemostasis. Inhibition of ADAMTS13 activity by autoantibodies causes immune thrombotic thrombocytopenic purpura (iTTP). Growing evidence has established allostery as a key contributor to iTTP pathophysiology. This review summarizes the current understanding of how ADAMTS13 structure contributes to its function and regulation and examines anti-ADAMTS13 antibodies as pathological allosteric modulators in iTTP and their associated allosteric mechanisms. **Methods**: We searched the published literature investigating the role of allostery in iTTP, with special emphasis on studies that satisfy the functional definition of allostery, which addresses how a ligand binding to a protein influences a second ligand-binding event at a distinct site. **Results**: Anti-ADAMTS13 antibodies primarily affect catalytic turnover as opposed to substrate binding affinity, consistent with their characterization as V-type allosteric ligands. Biophysical studies have revealed extensive and distal structural changes upon antibody binding, including near the enzyme’s active site. However, more recent work utilizing full-length Immunoglobulin G (IgG) molecules and polyclonal iTTP patient plasma suggests that the mechanistic complexity is greater than initially appreciated, with multiple mechanisms likely coexisting. **Conclusions**: While significant progress has been made to understand allostery in ADAMTS13 and iTTP, many questions remain unresolved. Further elucidation of these underlying mechanisms could help inform the development of diagnostic strategies and targeted therapies for this potentially fatal disorder.

## 1. Introduction

**Figure 1:**
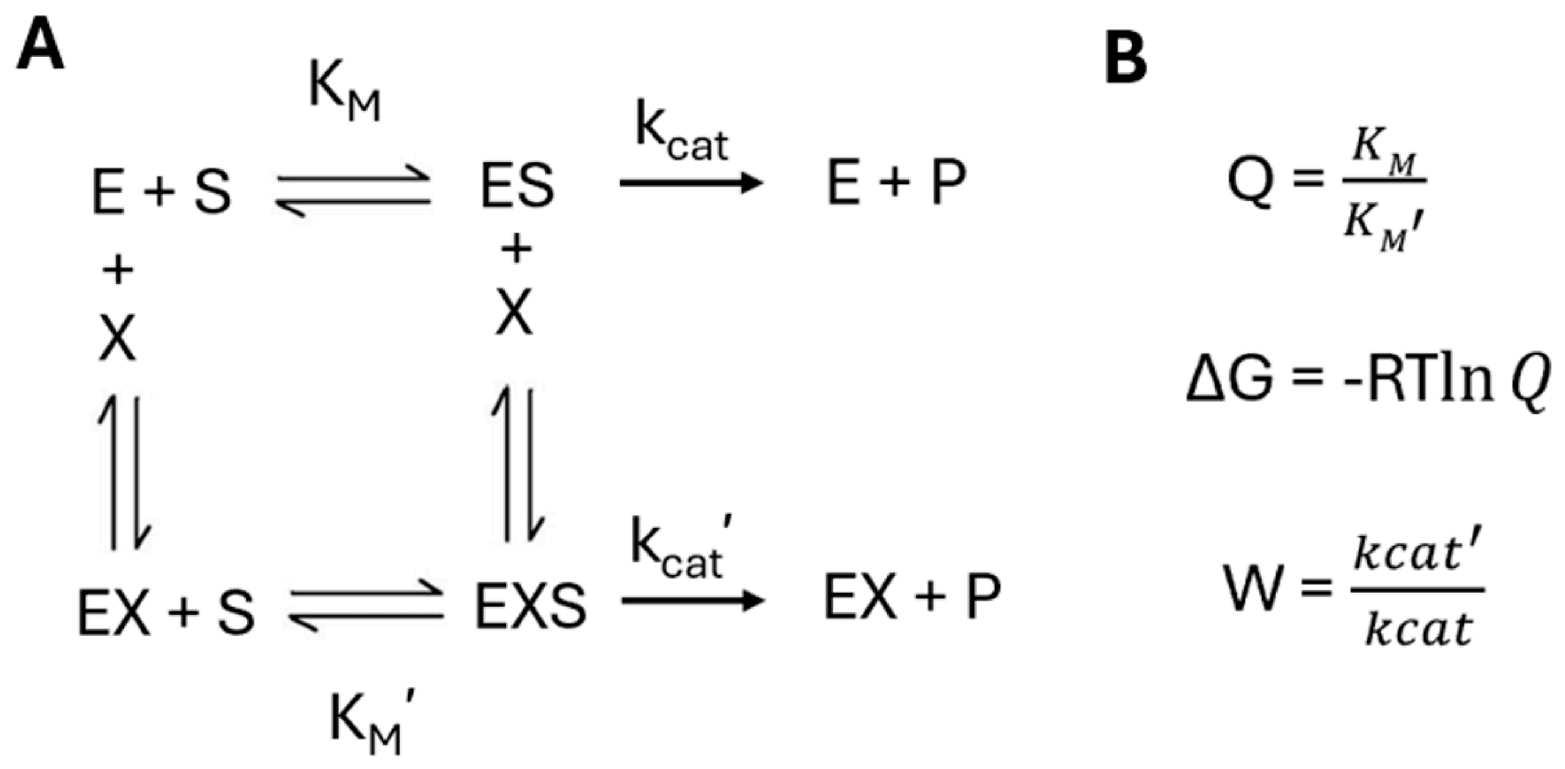
(**A**) Energy cycle depicting an allosteric mechanism involving an enzyme (E), substrate (S), and allosteric modulator (X). P denotes product. For the purposes of this review and in the context of immune TTP, E refers to ADAMTS13, S represents vWF, and X is an anti-ADAMTS13 antibody. (**B**) Equations showing how the thermodynamic parameters Q, ΔG, and W can be elucidated from the kinetic information indicated in panel A via the method of Reinhart. *K_M_* is the Michaelis constant, and *k_cat_* is the catalytic turnover rate. A prime symbol indicates that the parameter was derived in the presence of saturating concentrations of the allosteric ligand. Note that this is a simplified model, as in actuality, there are likely multiple antibodies bound to the enzyme, the substrate may bind at multiple places, and there could potentially be multiple subunits involved.

ADAMTS13 (A Disintegrin and Metalloprotease with Thrombospondin type 1 motifs, family member 13) is a plasma metalloprotease that plays an essential role in hemostasis through its cleavage of ultra-large multimers of von Willebrand Factor (vWF) [1]. ADAMTS13 also regulates the activity of vWF, since higher-molecular-weight vWF multimers are more hemostatically active than their lower-molecular-weight counterparts [2]. ADAMTS13 thereby prevents excessive platelet adhesion and thrombosis. This important function is compromised in immune thrombotic thrombocytopenic purpura (iTTP), a disorder in which ADAMTS13 activity is severely deficient due to the development of anti-ADAMTS13 autoantibodies [3]. The aberrant persistence of ultra-large vWF multimers results in severe thrombocytopenia and microangiopathic hemolytic anemia in these patients. Due to the potentially fatal consequences prompted by these autoantibodies, significant efforts have focused on their interaction with ADAMTS13 and their underlying mechanisms of inhibition. Evolving work has found that allostery plays an important role in the mechanism. In enzymology, allostery is defined as the binding of a ligand to a protein at one site (allosteric site) influencing a binding event of a second ligand at a distinct site (active site), typically through the induction of a conformational change. By this definition, allosteric systems must consider four protein complexes given the requirement of two separate binding events (Figure 1A) [4]. Generally, allosteric ligands can be characterized by how their presence modulates enzyme function, typically being classified as “K-type” if they alter substrate affinity or “V-type” if they influence catalysis [5]. However, despite representing a fundamental principle in protein regulation, our understanding of allostery and the mechanisms by which it occurs is still evolving. iTTP offers an exciting opportunity to explore allosteric systems and the phenomenon of allostery in disease biology. In this review, we will discuss the enzymology of ADAMTS13, the central role of allostery in the pathogenesis of immune thrombotic thrombocytopenic purpura (iTTP) and summarize emerging mechanistic insights.

## 2. Multidomain Organization and Conformational Regulation of ADAMTS13

**Figure 2:**
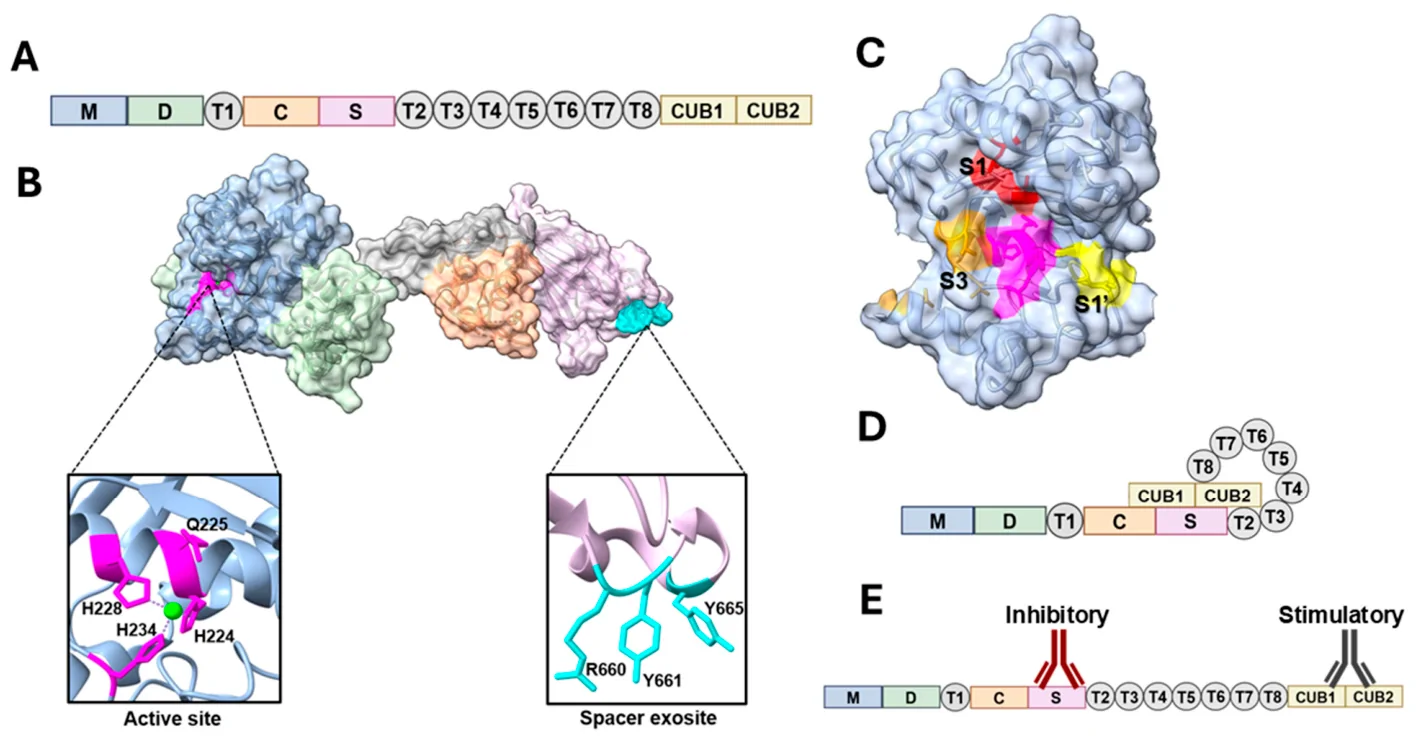
(**A**) Cartoon depicting ADAMTS13 and its domains. “M”—metalloprotease domain; “D”—disintegrin domain; “T1-8”—thrombospondin-like repeats; “C”—cysteine-rich domain; “S”—spacer domain. Two CUB domains are present at the enzyme’s C-terminal end. (**B**) Surface map of N-terminal domains with the active site (magenta) and spacer exosite (cyan) highlighted (PBD 6QIG). The catalytic zinc ion is represented by a green sphere. An E225Q substitution of the active site residue was made to allow for crystallization of the protein. (**C**) Metalloprotease domain with the active site colored magenta and the S1, S1′, and S3 binding pockets colored in red, yellow, and orange, respectively. (**D**) Schematic of the proposed spacer-CUB interaction responsible for global latency. (**E**) Commonly targeted exosites by anti-ADAMTS13 autoantibodies.

ADAMTS13 is a relatively large protein with a molecular weight of approximately 190 kDa, consisting of 14 domains. From the N- to the C-terminus, its full-length structure is comprised by a metalloprotease domain, a disintegrin-like domain, a thrombospondin-1 repeat (TSP1), a cysteine-rich domain, a spacer domain, seven additional thrombospondin repeats (TSP2-8), and two CUB (complement c1r/c1s, sea urchin epidermal growth factor, and bone morphogenetic protein) domains (Figure 2A) [6]. ADAMTS13 is primarily synthesized in hepatic stellate cells, then is secreted into the circulation. The high specificity ADAMTS13 has for vWF, which is its only known natural substrate to date, appears to be highly conformationally regulated [7]. vWF normally adopts a globular conformation in which its A2 domain, which contains the ADAMTS13 cleavage site, is inaccessible to the enzyme. In order for ADAMTS13-mediated cleavage to occur, vWF must first be unfolded by high shear stress of the flowing blood in the microvasculature, exposing the previously concealed A2 domain [8]. Likewise, the low promiscuity of ADAMTS13 has been attributed in part to a proposed intramolecular domain–domain interaction between the spacer and CUB domains that induces a “closed” conformation of the protein [9]. This global latency can be relieved *in vivo* by binding of the D4-CK domains of vWF to the ADAMTS13 CUB domains [10]. Once disrupted, several exosite interactions between vWF and ADAMTS13 take place along the length of the protein in a manner that has been deemed a “molecular zipper” [11]. These conformational changes position the Tyr1605-Met1606 bond correctly in the active site located in the ADAMTS13 metalloprotease domain. While these interactions do elicit critical structural changes, they do not fulfill the requirements to fit the classical definition of allostery that we have employed here for the purposes of this review (i.e., not a four-complex thermodynamic cycle). Furthermore, we acknowledge that the binary concept that ADAMTS13 either exists in a “closed” or “open” conformation is likely an oversimplification of a complex, dynamic protein capable of adopting multiple conformational states. Nonetheless, it is clear that there exists long-range communication across the large, multidomain structure of ADAMTS13 relating to its function and regulation, even in physiological contexts.

## 3. Exosite Interactions Are Vital to the Proteolytic Function of ADAMTS13

### 3.1. Metalloprotease Domain

The metalloprotease domain of ADAMTS13 contains the enzyme’s active site (Figure 2B). Typical of the metzincin protein family, this domain contains the conserved zinc-binding motif, HEXXHXXGXXHD, and the characteristic Met-turn motif (Met249) [12]. Within the catalytic cleft, His224, His228, and His234 coordinate the essential Zn^2+^ ion, and the active site residue, Glu225, helps to activate water to act as a nucleophile for hydrolysis of the peptide bond in vWF [13]. Structural studies have identified several subsites surrounding the active site as being important for substrate recognition and successful proteolysis [14]. The S1 pocket in the metalloprotease domain involving Leu151 and Val195 accommodates the P1 residue (Tyr1605) of vWF. Meanwhile, the metalloprotease residues Asp252, Gly253, Ala254, Ala255, and Pro256 form the S1′ subsite responsible for binding Met1606 of vWF. The P3 residue of vWF, Leu1603, has been found to be highly important for ADAMTS13-mediated proteolysis, as substitution at this site results in significantly reduced catalytic efficiency [15]. The corresponding S3 pocket that has been hypothesized to interact with Leu1603 consists of Leu198, Leu232, and Leu274 in the ADAMTS13 metalloprotease domain (Figure 2C). Despite being the site of catalysis, the metalloprotease domain alone is not sufficient for ADAMTS13′s cleavage activity [16]. Furthermore, the minimal structure of vWF that can be cleaved consists of 73 amino acids within the VWF A2 domain [17]. Additional domains must interact with vWF to enable proper proteolysis. We will further discuss some of these other important exosite interactions in the following sections.

### 3.2. Spacer Domain

The presence of the ADAMTS13 spacer domain is necessary for the full proteolytic function of the enzyme, and it serves as a major exosite for vWF recognition [16,18]. The spacer domain interacts with the C-terminal portion of the vWF A2 domain, particularly residues Glu1660-Arg1668 [19]. Disruption of this spacer-binding site in VWF results in impaired proteolysis, mainly resulting from an increase in *K_M_*, indicating that this exosite is primarily implicated in conferring substrate binding affinity [20]. The spacer domain residues Arg660, Arg661, and Tyr665 have been shown to be critical for substrate recognition and subsequent cleavage by ADAMTS13 (Figure 2B) [21]. The importance of the spacer domain for vWF scissile bond cleavage is an intriguing observation, given that the spacer domain lies approximately 40 Å (and ~300 amino acids) distal to the metalloprotease domain, which is the location of the active site [20]. However, a sophisticated and thorough study that integrated several biochemical and biophysical techniques supports a model in which the TSP1 domain acts as a flexible hinge to bring the spacer domain in close proximity to the metalloprotease domain [22].

### 3.3. CUB Domains

ADAMTS13 contains two CUB domains at its C-terminal end and is interestingly the only ADAMTS family member to possess them. Compared to the wild-type full-length protein, ADAMTS13 truncated after the spacer domain (MDTCS) exhibits ~2.5X higher activity towards cleaving synthetic vWF peptidyl substrates [23]. This observation indicated that the C-terminal domains are dispensable for proteolytic activity and may play a regulatory role via autoinhibition. Additionally, binding studies performed by Zanardelli et al. suggested that ADAMTS13 can bind to globular vWF at a constitutively exposed site on the surface of the D4-CK domains, and this binding is mediated by the distal TSP5-CUB region of ADAMTS13 [24]. Thus, the ADAMTS13 CUB domains may be involved in the first step of a proposed multi-step interaction model that allows for docking and exposure of sites that were previously hidden in folded vWF.

In 2021, the crystal structure of the ADAMTS13 CUB domains was resolved, providing new insights into the structure and potential contributions of this region of the enzyme [25]. While this structure exhibited similarities with other tandem CUB domains, several key features were observed that make the ADAMTS13 CUB domains distinctive, including a much larger interface between CUB1 and CUB2 (758 Å^2^) and the lack of a typically conserved Ca^2+^ binding site. Furthermore, the resolved structure was used to investigate the mechanism underlying the autoinhibitory effect of the CUB domains. Previous studies have suggested that an intramolecular interaction between the CUB domains and the proximal domains is responsible for the “global latency” of ADAMTS13 by promoting a closed conformation that is less active (Figure 2D). The CUB domains have been proposed to interact with the spacer domain in particular, as substitution of certain spacer domain residues abrogated the inhibitory effect of isolated, purified CUB domains on activity of MDTCS [23,26,27]. Through a mutagenesis panel and *in silico* docking simulations, the authors proposed a model of the spacer–CUB interaction in which Glu634 and Asp635 form salt bridges with Lys1252 and Arg1272 in CUB1. At the center of the interface, the hydrophobic patch consisting of Leu591 and Phe592, Leu637, and Leu668 interacts hydrophobically with Trp1245, Leu1248, and Trp1250 in CUB1. The spacer domain residues Arg660-Try665, which have been implicated in vWF binding, are accommodated ionically by the pocket formed by Arg1326, Arg1361, Glu1387, and Glu1389 in CUB2 [25]. Thus, it has been postulated that *in vivo*, the ADAMTS13 CUB domains bind to vWF D4-CK, which disrupts the spacer–CUB interface and opens up vWF to expose cryptic sites that can now engage with the spacer domain and subsequent proximal domains to help orient the Tyr1605-Met1606 bond correctly in the active site cleft of the metalloprotease domain.

## 4. ADAMTS13 May Form Functional Multimers

Evidently, intermolecular interactions between ADAMTS13 and vWF are critical for protein function, and intramolecular domain-domain interactions are important in its regulation. Intriguingly, there has been recent accumulating evidence that there may exist intermolecular interactions between ADAMTS13 monomers that could have significant ramifications on its function and pathological inhibition in the context of iTTP. More specifically, a study by Rottensteiner et al. observed that ADAMTS13 had the propensity to form homo-oligomers seemingly through covalent disulfide bond formation [28]. In a recent study published by our group, there was strong positive cooperativity between two patient-derived anti-ADAMTS13 antibody fragments when titrated under physiological pH and temperature conditions [29]. The Hill coefficient fitted from a four-parameter nonlinear regression analysis was comparable to that for hemoglobin, the classic example of how cooperativity applies to a biological framework. In conjunction with the study that showed ADAMTS13 can multimerize, this finding suggests that ADAMTS13 may form multimers that are functional, as one potential hypothesis that supports the observed phenomenon is that binding of an antibody to one monomer of ADAMTS13 may increase the likelihood that another antibody binds to an adjacent monomer. Alternatively, the positive cooperativity observed may be more akin to a glucokinase-like mechanism in which a monomer can adapt multiple stable intermediate conformations. However, whether ADAMTS13 forms multimers *in vivo* and the physiological relevance of such complexes, if any, is unknown and requires further investigation. Nevertheless, the positive cooperativity between the two antibodies implied that allosteric effects may contribute to the behavior of the system, which we will discuss in more detail in the upcoming sections.

## 5. Anti-ADAMTS13 Autoantibodies Are Developed in Immune Thrombotic Thrombocytopenic Purpura (iTTP)

In contrast to the rarer congenital form of TTP (cTTP), the severe deficiency of ADAMTS13 activity in iTTP results from anti-ADAMTS13 autoantibodies [3,30]. These autoantibodies are primarily IgG, although IgA and IgM isotypes have also been reported. Of the IgG subtypes, IgG_1_ and IgG_4_ are the most prevalent in these patients [31]. Whereas IgG_1_ antibodies are bivalent, the IgG_4_ antibodies likely circulate *in vivo* as functionally monovalent ligands given their tendency to undergo Fab arm exchange (FAE) [32]. Epitope mapping studies have demonstrated that the spacer domain is the most frequently targeted exosite, with essentially all patients harboring inhibitory anti-spacer antibodies [33,34,35]. However, the autoimmune response in iTTP is polyclonal, and all the domains of ADAMTS13 have been identified as being targeted [36]. Interestingly, a significant portion of patients (around 30–50%) possess anti-CUB antibodies (Figure 2E). The antibodies targeting this region have been shown to stimulate ADAMTS13 *in vitro*, and their role in iTTP pathophysiology remains elusive, with some suggesting that they may promote clearance of ADAMTS13, disrupt the spacer-CUB interaction to expose the spacer domain for immune targeting, or impact the interaction between the CUB domains and vWF D4-CK [37,38,39]. Since it has been shown that the spacer domain is also critical for vWF recognition and engagement, the prevailing hypothesis in the field for many years was that inhibitory anti-ADAMTS13 antibodies impair enzyme function through steric interference with substrate binding. In the next section, we will delve into how this hypothesis was tested, the surprising results, and the work that has been done up to this point to better understand the allosteric mechanisms at play.

## 6. Allosteric Effects of Anti-ADAMTS13 Antibodies

The mechanistic effects of anti-ADAMTS13 antibodies and the role of allostery have recently become an area of active investigation, with most studies being confined to the past decade. Because the spacer domain is the primary immunologic target, much of this work has focused on anti-spacer antibodies. A study employed hydrogen deuterium exchange mass spectrometry (HX-MS) in the presence and absence of three anti-spacer single-chain fragments of the variable regions (scFvs) that had been isolated from iTTP patients by phage display [33,40]. The results showed that all three antibodies recognized the same discontinuous epitope consisting of five solvent-exposed loops in the spacer domain. Deletion of any one of the loops resulted in loss of antibody binding but also a significant impairment of ADAMTS13 activity. Furthermore, one of the identified loops consisted of the spacer domain residues spanning positions 659–664; this motif includes Arg660, Arg661, and Tyr665, the residues that have been found to be vital for vWF binding and cleavage [21]. Thus, these findings seemed to support the predicted inhibitory mechanism involving physical blocking of the substrate. However, in 2020, a study was published that analyzed the effects of monoclonal murine anti-human anti-ADAMTS13 antibodies on the kinetics of proteolysis [41]. Contrary to the expected outcome, the results demonstrated that the antibodies mainly affected catalytic turnover (*k_cat_*) as opposed to substrate concentration-dependent effects on velocity (*K_M_*). When ADAMTS13 was incubated with the antibodies, a normally cryptic site in the metalloprotease domain was exposed, allowing for capture by the anti-metalloprotease 6A6 antibody in an ELISA assay. Together, these data supported the idea that anti-ADAMTS13 antibodies elicit conformational changes that alter the catalytic efficiency of the enzyme rather than the ability of vWF to bind, pointing to a potential allosteric mechanism.

**Figure 3:**
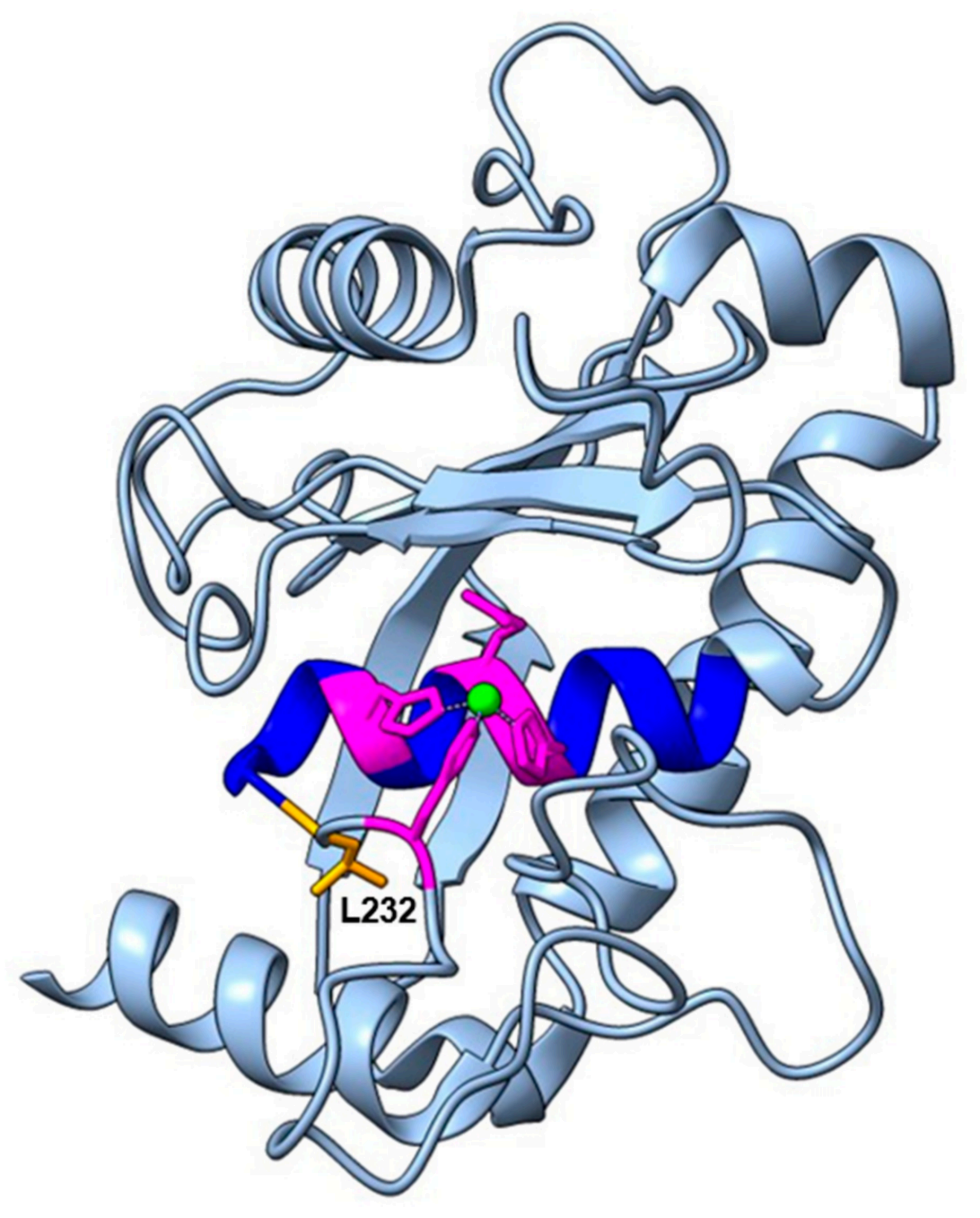
Cartoon structure of ADAMTS13 metalloprotease domain with the catalytic zinc ion represented by a green sphere and active site residues in magenta (PBD 6QIG). The binding of three anti-spacer antibody fragments induced a common pattern of conformational changes near the active site, evidenced by increased deuterium uptake in the α4 helix (dark blue) and in the immediately distal region containing the L232 residue (orange).

Our group sought to investigate if human monoclonal antibodies would yield similar results to the reported murine antibodies. Using the same three anti-spacer scFvs utilized in the aforementioned HX-MS study, we performed enzyme kinetic analyses and observed that the presence of these antibodies significantly decreased the maximal velocity (*V_max_*) while having little impact on *K*_0.5_, a more generalizable term to describe the substrate concentration at half-maximal velocity that is not as restricted to assumptions of Michaelis-Menten kinetics [42]. These results indicate that inhibition of ADAMTS13 by antibodies may occur through altering the rate of catalytic turnover without significantly impacting substrate recognition, consistent with the results seen when using the mouse monoclonal antibody panel. Complementary evidence obtained through HX-MS experiments demonstrated that binding of the inhibitors at the spacer domain resulted in conformational changes near the active site in the distal metalloprotease domain (Figure 3). The observed pattern of increased deuterium uptake is suggestive of unfolding of the α4 helix, which contains several of the residues that make up the protease’s active site. Disruption of the secondary structure around this site could modify the enzyme’s ability to coordinate the essential Zn^2+^ ion. Furthermore, differential deuterium uptake was observed immediately distal to this α4 helix where the L232 residue is located. L232 has been implicated in forming the S3 pocket that binds L1603 on vWF; therefore, anti-spacer antibody binding may affect vWF binding and accommodation near the catalytic cleft.

Due to the observed conformational changes and indications towards an allosteric mechanism, we previously conducted a thermodynamic analysis by applying the method of Reinhart to quantitatively determine the linkage between antibody and substrate binding [43]. According to this method, the free energy of interaction (ΔG) between an allosteric ligand and the substrate can be obtained from the parameter Q, which is derived from the ratio of the substrate concentration at half-maximal activity in the absence (*K*_0.5_) and presence of saturating concentrations of ligand (*K*_0.5_′) (Figure 1B) [44]. The data showed that two anti-CUB scFvs produced Q values close to 1, resulting in ΔG values near zero, meaning that the thermodynamic effects of such antibodies on substrate binding are essentially negligible. In contrast, when comparing maximal velocity in the absence (*V_max_*) and presence (*V_max_*′) of saturating antibody, expressed as the ratio W (Figure 1B), the derived W parameters were greater than 1, signifying that the presence of these antibodies significantly increased *V_max_* compared to when no antibody was added. Overall, this thermodynamic analysis further supported the idea that anti-ADAMTS13 antibodies are V-type ligands, primarily affecting *V_max_* while having little to no impact on substrate binding.

Nearly all the research conducted to this point had utilized either scFv or murine antibodies to investigate the mechanistic effects of autoantibodies in iTTP. To more closely mimic the *in vivo* pathophysiology, a recent study generated full-length human IgG constructs with the same complementarity determining regions (CDRs) as previously characterized anti-ADAMTS13 scFvs [45]. IgG_1_ and IgG_4_ were engineered, as these represent the most prevalent subclasses in iTTP patients. Interestingly, in contrast to the corresponding scFvs, results with the IgGs exhibited varied mechanisms depending on where they bind to the enzyme and the reaction condition used. More specifically, all IgGs tested displayed V-type behavior in a condition closer to physiologic pH and temperature (pH 7.0, 37 °C). However, under a clinical diagnostic condition (pH 6.0, 25 °C), the anti-spacer inhibitory IgGs shifted to more K-type behavior, while stimulatory IgGs directed against the C-terminal end of ADAMTS13 were observed to produce a similar effect at pH 7.0 and 25 °C. Regardless of condition, no subclass-specific differences were detected between IgG_1_ and IgG_4_ constructs with the same CDR sequence. Furthermore, to better recapitulate the polyclonal nature of the disease, the investigators performed similar experiments with iTTP patient plasma and observed variability in mechanisms between patients in both a diagnostic and a more physiologic assay condition. Ongoing work will determine whether the variability in mechanistic behavior correlates with clinical outcomes. Together, these findings indicate that antibody-mediated modulation of ADAMTS13 in iTTP likely occurs via multiple mechanisms with both thermodynamic and kinetic regulation involved, especially under non-physiological pH and temperature.

## 7. Discussion

Immune TTP is a life-threatening thrombotic microangiopathy caused by anti-ADAMTS13 autoantibodies that impair the enzyme’s ability to sufficiently proteolyze von Willebrand Factor [46]. Over the last 10 years, a growing body of work has revealed that allostery plays a central role in this disease process. The finding that anti-ADAMTS13 antibodies primarily function as V-type allosteric ligands represented a paradigm shift in the field, challenging the long-standing assumption that autoantibodies most likely act through direct blockage of substrate binding at the spacer domain. However, as the research has progressed, the mechanistic landscape has become increasingly complex. Recent evidence suggests that pathological antibodies may inhibit ADAMTS13 through a diversity of mechanisms, including allosteric effects that disrupt the catalytic efficiency of the distant active site and altered substrate binding, and these mechanisms appear to differ among patients.

Despite significant progress being made, the work encompassing allostery involving ADAMTS13 is nascent, and further elucidation of its mechanisms is required. One important area for future research is the avidity effects of anti-ADAMTS13 antibodies. In antibody biology, avidity represents one of the fundamental determinants of antibody function [47]. This line of investigation is especially compelling in regard to iTTP given the prevalence of the IgG_4_ subclass. Whereas a typical IgG possesses two Fab arms capable of binding two ADAMTS13 simultaneously, IgG_4_ antibodies typically exchange a Fab arm with IgG_4_ antibodies of unrelated specificity *in vivo*. Thus, through this process, they become bispecific, functionally monovalent molecules that are largely incapable of avidity-driven interactions. This phenomenon has been shown to contribute to pathogenic mechanisms in other IgG_4_-mediated autoimmune diseases, but its involvement in iTTP pathogenesis, if any, is unknown [47]. Therefore, comparisons between bispecific and divalent antibodies directed against ADAMTS13 could provide interesting insights that are physiologically relevant. Along with avidity, other antibody subclass-specific differences, such as effector functions and propensity for complement pathway activation, may influence the pathogenicity of anti-ADAMTS13 antibodies and warrant future investigation. Intriguingly, the observed cooperativity between two anti-ADAMTS13 antibodies raises the possibility that ADAMTS13 forms functional multimers, suggesting that intermolecular domain–domain interactions between different ADAMTS13 monomers may add an additional layer of allosteric regulation that could impact both ADAMTS13 function and antibody-mediated inhibition.

Importantly, advancing our mechanistic understanding of anti-ADAMTS13 antibodies can have significant clinical implications. The source of disease heterogeneity observed among iTTP patients remains unclear. Several groups have sought to establish a correlation between anti-ADAMTS13 autoantibodies and disease severity [48,49,50]. Some studies suggest that a higher antibody titer is associated with worse treatment responses and higher mortality, burden of care, and risk of recurrence [51,52,53,54,55]. However, other studies were unable to validate such a correlation, making the prognostic value of anti-ADAMTS13 antibodies a currently controversial matter [56]. As indicated by the evidence discussed in this review, functional antibody characteristics, including target epitopes of inhibitory antibodies, mechanism of action, and whether one or more monoclonal antibodies in a polyclonal milieu act as drivers, may contribute substantially to pathophysiologic variability. Thus, studying qualitative properties of a patient’s antibody profile, such as their capacity to exert allosteric effects, could be of greater clinical benefit than simply analyzing the overall antibody quantitation, as conventional laboratory methods have focused upon. Still, several important questions remain unanswered, and future work will be needed to determine the role of antibody mechanisms on outcomes in a clinical setting.

Consideration of anti-ADAMTS13 antibodies as allosteric ligands can also reframe how we approach iTTP treatment. The current standard of care for patients is therapeutic plasma exchange (TPE), which removes the circulating autoantibodies and immune complexes while simultaneously replenishing ADAMTS13. Immunosuppressive therapies, such as general B-lymphocyte depletion with the anti-CD20 monoclonal antibody rituximab, are often employed in the treatment course to prevent autoantibody production. More recently, caplacizumab has become a first-line therapy for iTTP, functioning to inhibit the vWF-platelet interaction by competing for the same binding site at the vWF A1 domain as the platelet GPIb receptor [46]. While these treatment strategies address disease aspects, none are designed to specifically prevent or reverse the allosteric modulation on ADAMTS13 induced by the anti-ADAMTS13 antibody population. As our understanding of allosteric mechanisms in iTTP continues to evolve, these lessons can inform the development of new therapeutic interventions that address the underlying pathophysiology more directly. ADAMTS13 variants designed to resist antibody-mediated allosteric effects or agents that allosterically stabilize favorable conformational states could allow for more precise and effective treatment options. Determining patient-specific antibody profiles may become increasingly important in clinical management. Furthermore, as the field of allosteric drug design continues to gain interest and traction, iTTP could serve as a model system to increase our understanding of allosteric regulation and how it can be leveraged in the therapeutic design space. In this way, the research of allostery in ADAMTS13 can expand to benefit a broad range of diseases and provide insights into the fundamental principles of allosteric regulation that govern protein function across biology.

## Data Availability

No new data were created or analyzed in this study. Data sharing is not applicable to this article.

## Abbreviations

The following abbreviations are used in this manuscript:

iTTP	Immune thrombotic thrombocytopenic purpura
ADAMTS	A Disintegrin and Metalloprotease with Thrombospondin type 1 motifs
vWF	Von Willebrand Factor
scFv	Single-chain fragment of the variable region
IgG	Immunoglobulin G
CDR	Complementarity determining region
FAE	Fab arm exchange
